# Gut Microbial Translocation in Critically Ill Children and Effects of Supplementation with Pre- and Pro Biotics

**DOI:** 10.1155/2012/151393

**Published:** 2012-08-15

**Authors:** Paola Papoff, Giancarlo Ceccarelli, Gabriella d'Ettorre, Carla Cerasaro, Elena Caresta, Fabio Midulla, Corrado Moretti

**Affiliations:** ^1^Pediatric Emergency and Intensive Care Division, Department of Pediatrics, Sapienza University of Rome, Viale Regina Elena 324, 00161 Rome, Italy; ^2^Department of Public Health and Infectious Diseases, Sapienza University of Rome, 100161 Rome, Italy

## Abstract

Bacterial translocation as a direct cause of sepsis is an attractive hypothesis that presupposes that in specific situations bacteria cross the intestinal barrier, enter the systemic circulation, and cause a systemic inflammatory response syndrome. Critically ill children are at increased risk for bacterial translocation, particularly in the early postnatal age. Predisposing factors include intestinal obstruction, obstructive jaundice, intra-abdominal hypertension, intestinal ischemia/reperfusion injury and secondary ileus, and immaturity of the intestinal barrier per se. Despite good evidence from experimental studies to support the theory of bacterial translocation as a cause of sepsis, there is little evidence in human studies to confirm that translocation is directly correlated to bloodstream infections in critically ill children. This paper provides an overview of the gut microflora and its significance, a focus on the mechanisms employed by bacteria to gain access to the systemic circulation, and how critical illness creates a hostile environment in the gut and alters the microflora favoring the growth of pathogens that promote bacterial translocation. It also covers treatment with pre- and pro biotics during critical illness to restore the balance of microbial communities in a beneficial way with positive effects on intestinal permeability and bacterial translocation.

## 1. Introduction

Despite advances in diagnosis and treatment, bacterial sepsis remains a significant cause of pediatric morbidity and mortality, particularly among critically ill children. Sepsis is the consequence of microbial invasion, or microbial products release, into the bloodstream, which result in systemic inflammatory response syndrome (SIRS). Bloodstream infection may arise through multiple routes, including bacterial translocation across the epithelial-mucosa as in the airways, gastrointestinal tract, kidney or genital tract, and skin breaks as in wounds and during insertion of central venous catheters or other medical devices [1]. Among these different possibilities, bacterial translocation across the gastrointestinal tract has been suggested as one of the principal pathogenetic mechanisms of sepsis and organ dysfunction among critically ill children [2]. There are a number of reasons why bacterial translocation may be relevant to the development of sepsis in children requiring intensive care: the majority of infections diagnosed in children in intensive care unit are due to microorganisms already present in the patients' admission flora of the throat and gut [3]; critical illness, coupled with intensive care treatment, results in a high reduction in microbiota biodiversity with a massive increase of enterococci [4]; gut permeability alterations to large molecules have been documented in critically ill patients [5]; selective gut decontamination seems to reduce infections in a subset of patients admitted to intensive care unit [6]; early enteral feeding is associated with reduced incidence of infections in critically ill patients [7]. Although clinical evidence suggests the importance of the gastrointestinal tract in the development of sepsis syndrome, bacterial translocation itself may not be the primary cause [8].

The purpose of this paper is, therefore, to discuss bacterial translocation, including its definition and role in causing sepsis syndrome and organ failure in critically ill children. The present paper also includes an analysis of the potential benefits of prebiotic and probiotics supplementation for the prevention of sepsis.

## 2. Gut Microflora and Its Significance

Human gut contains ~500 different species of microbes as commensals, including obligate anaerobes (about 95%) and facultative anaerobes (1–10%). Obligate anaerobes include *Bifidobacterium*, *Clostridium*, *Eubacterium*, *Fusobacterium*, *Peptococcus*, *Peptostreptococcus,* and *Bacteroides*, and facultative anaerobes *Lactobacillus*, *Bacillus*, *Streptococcus*, *Staphylococcus*, *E. coli*, *Klebsiella*, and *P. aeruginosa*. Bifidobacteria are the predominant microbes representing up to 80% of the cultivable fecal bacteria in infants and 25% in adults [9]. Although commensal bacteria are present in extremely high numbers, they rarely cause local or systemic disease, while have several important physiologic effects in the distal intestinal tract [10]. They directly activate the development and differentiation of the intestinal epithelium and its immune system and contribute to maintain an immunologically balanced inflammatory response. Microflora has nutritive functions, as well. It produces several enzymes for fermentation of nondigestible dietary residue and for secretion of endogenous mucus and helps in recovering lost energy in the form of short-chain fatty acids. It also plays a part in synthesis of vitamins, and in absorption of calcium, magnesium, and iron [11]. Finally, the gut microflora provides a physical barrier against invading pathogens, the so-called “colonization resistance” [12], through a competition for epithelial cell adhesion sites and available nutrients, and by releasing antibacterial substances (e.g., bacteriocins and lactic acid).

Prolonged critical care therapy may disrupt the balance between the host and the gut commensal flora in several ways [13]. Virtually all critically ill patients receive antibiotics which may profoundly affect the intestinal commensal microflora. Using molecular biology techniques, Iapichino et al. evaluated the intestinal microbiota composition of previously healthy patients on admission to intensive care unit [4]. While the first faecal samples showed a banding pattern that was similar to that of healthy subjects, after one week of critical illness and intensive care treatment, including antibiotics, a well definite alteration in the overall microbiota composition was evident, with the presence of a dominant band related to *Enterococcus*. An alteration of intestinal microbial flora has also been observed in premature neonates admitted to intensive care unit. Due to widespread use of broad spectrum antibiotics and late feeding, newborn infants present a delayed colonization with Lactobacillus and Bifidobacteria and a rapid appearance of enterococci, including *Enterococcus faecalis*, *E. coli*, *Enterobacter cloacae*, and the *Klebsiella pneumonia* [14]. Unexpectedly, *C. difficile* colonization does not increase in the early neonatal period despite the use of antibiotics such as cephalosporins [15]. Ferraris et al., who investigated the incidence and perinatal determinants of clostridial colonization in premature neonates, have recently confirmed the absence of effect of either antenatal or postnatal antibiotics on the overall clostridial colonization in neonates admitted to intensive care [16]. In the absence of antibiotics to disrupt the microbiota, it is not clear which event precedes *C. difficile* proliferation. It has been speculated that a decrease in *Bacteroides* and an increase in facultative anaerobes might facilitate colonization by *C. difficile* without a need for the action of antibiotics [17]. The influence of antibiotic use may be more relevant in affecting the severity of *Clostridium difficile* infection as suggested by the results of a recent study by Kim et al. showing that the most significant risk factors for severe *C. difficile* disease in children included young age (adjusted odds ratio [95% confidence interval]: 1.12 [1.02; 1.24]) and receipt of 3 antibiotic classes in the 30 days before infection (3.95 [1.19; 13.11]) [18]. In addition to antibiotic use, several other factors may predispose the gut ecology to alterations during the care of critically ill children. The use of gastroprotectant agents favors proliferation of acid sensitive organisms within the upper intestinal tract. Vasoactive agents that severely limit mesenteric perfusion induce profound luminal hypoxia and hypercarbia that are potent activators of bacterial virulence gene expression [19, 20]. The use of opioids as sedative analgesic agents in mechanically ventilated patients create intestinal atonia and bacterial overgrowth [21]. Finally, nutrients delivered to critically ill patients intravenously or as highly processed foods, whose absorption is nearly complete within the small intestine, create nutrient scarcity within the distal intestinal tract, the area where the highest microbial burden exists. Under such circumstances, the proliferation of highly virulent microorganisms creates a state of perturbed host pathogen balance, and an undesired activation of local inflammation. Systemic inflammation can thus be initiated by mucosally derived cytokines, or when microbial products enter the systemic circulation through the disrupted intestinal epithelial barrier [22].

## 3. Concepts of Bacterial Translocation

Bacterial translocation has been defined as the process by which live bacteria, their products, or both cross the intestinal barrier where they may either directly cause infection or excite the immune system resulting in a massive inflammatory reaction causing diffuse organ damage and eventually organ failure and death [23]. To some extent, bacterial translocation from the gut occurs regularly even in healthy individuals (5–10%) [24], but bacteremia is generally limited by an intact immune system [8]. This may be a normal physiologic process by which animals and humans sample different luminal antigens in order to produce immunocompetent cells [25]. The first line of defense for preventing bacterial translocation is the mucous coat overlying gut epithelia, produced by goblet cells, which includes degraded mucin and antimicrobial peptides. In the neonatal enterocytes, the ability of mucin to inhibit bacterial translocation may be diminished compared to adults [26]. This might help to explain the propensity of the neonatal rats to spontaneous bacterial translocation in the first two weeks of life when intestinal concentrations of gram-negative bacilli and gram-positive cocci are high, and the concentration of lactobacilli is low [27]. Translocation occurs in between cells or through cells of the intestinal epithelium after loss of tight junctions between enterocytes. Several mechanisms of bacterial translocation have been identified from studies of enteropathogenic bacteria, such as a zipper mechanism that utilizes transmembrane cell-adhesion proteins as receptors for the bacteria [28]; a bacterial needle-like probe that injects dedicated bacterial effectors into epithelia and the injected molecules modify the cytoskeleton to facilitate bacterial entry [29]; an increased nitric oxide production during inflammatory states that alters expression and localization of the tight junction proteins that surround the upper part and lateral surfaces of enterocytes leading to intestinal hyperpermeability [30]; toll-like receptors that are present on the luminal surface of enterocytes to sense danger and activate host defenses and that can also be harmful by mediating phagocytosis and translocation of bacteria across the intestinal barrier [31]. Once pathogens pass the mucus and epithelial barriers, they are ingested by submucosal macrophages. This process occurs without initiation of an inflammatory response [32]. If intestinal macrophages are dysfunctional as in very low-birth-weight infants [33, 34], this dysfunction may contribute to diffusion of bacteria in the systemic circulation.

Several factors may enhance bacterial translocation in critically ill children. Bacterial overgrowth and breakdown of a tight junction in the setting of intestinal obstruction has been shown to promote bacterial translocation in animal models [35] and in humans [36]. There is evidence in *in vitro* and animal studies that obstructive jaundice impairs reticuloendothelial function [37], interferes with macrophage activation [38], alters Kupffer cell function [39], promotes disruption of desmosomes and formation of lateral spaces between enterocytes [40], and, therefore, alters epithelial barrier permeability. Another possible mechanism for increased translocation associated with obstructive jaundice is considered to be the inhibitory effect of bile on bacterial invasion of enterocytes shown *in vitro* [41]. Intra-abdominal hypertension and abdominal compartment syndrome may also cause gut barrier dysfunction [42]. Another factor that promotes bacterial translocation and predisposes to development of SIRS and organ dysfunction is intestinal ischemia/reperfusion injury. The gut is an organ extremely sensitive to systemic cardiovascular and pulmonary disturbances. The physiological response to hypoperfusion is the shunting of blood away from splanchnic circulation toward more vital organs. The consequence is ischemia/reperfusion injury of villi, release of proinflammatory factors, mucosal disruption, increased intestinal permeability, and bacterial translocation. In addition, secondary ileus seen after ischemia/reperfusion injury seems to promote bacterial overgrowth and proximal gut colonization which are linked with the development of septic complication [43].

Among the factors that influence bacterial translocation, postnatal age and prematurity appear to play a significant predisposing role. Prematurity reduces mucosal barrier function and consequently foster gut permeability [20]. Moy gave definitive experimental evidence that spontaneous bacterial translocation occurs in the neonate by demonstrating that transformed *E. coli* K1 fed to healthy rabbit pups spontaneously translocated from the intestinal lumen and subsequently disseminated to the mesenteric lymph nodes, spleen, and liver [44]. A high proportion of bacterial translocation in neonates results not only from immaturity of host defense functions, but also from the dominant colonization of aerobic bacteria in the intestine. Bacterial colonization develops differently in breast-fed, formula-fed, premature, and full-term infants. In a model of newborn rats, Yajima showed that breastfeeding inhibited systemic bacterial translocation in the suckling period of the rat, even though this phenomenon is not necessarily correlated to modification of the colonizing flora [24].

There are precise criteria to define that bacterial translocation has occurred in a subject including: gut-origin bacteria found in mesenteric lymph nodes (the first organ encountered by the organism undergoing translocation) or portal venous blood; endotoxin found in mesenteric lymph nodes or portal venous blood; bacterial DNA or proteins found in mesenteric lymph nodes, portal venous blood, or the systemic circulation; development of infectious complications with organisms that presumably originated from the gut; increased levels of circulating and tissue cytokines and inflammatory mediators; increased permeability of the gut to large molecules. Increased intestinal permeability as measured by the lactulose-to-mannitol ratio may be a permissive factor for bacterial translocation, but finding an increased lactulose-to-mannitol ratio does not prove that translocation has occurred [8]. In humans in whom direct culture of mesenteric lymph nodes or portal blood is not routinely possible, we often use indirect ways to confirm or monitor bacterial translocation, thus extreme caution should be practiced when drawing conclusions.

## 4. Bacterial Translocation in Critically Ill Patients

Few studies have investigated the pathogenic potential of bacterial translocation to septic morbidity in critically ill children. Pathan et al. examined the role of intestinal injury and subsequent endotoxemia in the pathogenesis of organ dysfunction after surgery for congenital heart disease [45]. They analyzed blood levels of endotoxin alongside global transcriptomic profiling and monocyte endotoxin receptor expression in children undergoing surgery for congenital heart disease and found that these infants present an increased risk of intestinal mucosal injury and endotoxemia which may contribute to inflammatory activation and organ dysfunction postoperatively. Cicalese et al. evaluated the correlation between bacterial translocation and preservation injury or acute rejection in 50 pediatric small bowel transplant immunosuppressed recipients [46]. Bacterial translocation episodes were considered when microorganisms were found simultaneously in blood or liver biopsy and stool. In this study, a substantial percentage of bacterial translocation was associated with acute rejection, the presence of a colon allograft, and a long cold ischemia time. In a prospective observational cohort study in 94 neonates and infants who underwent surgical procedures and required parenteral nutrition because of gastrointestinal abnormalities, Pierro et al. explored the relevance of septicemias due to microbial translocation in relation to long-term parenteral nutrition [47]. Microbial translocation was diagnosed when the microorganisms that were isolated from the blood sample were also carried in the throat and/or rectum. Sepsis associated with microbial translocation was found in 15 cases on 6 infants. *Escherichia coli*, *Klebsiella*, *Candida* species, and enterococci were more commonly isolated. The authors concluded that in neonates and infants who are receiving parenteral nutrition, septicemia may be a gut-related phenomenon. From the results of these studies, it appears that bacterial translocation could indeed be a critical component to the development of SIRS; however, the numerous methodological problems that plague these studies make a cause/effect relationship questionable.

## 5. Prebiotics 

The introduction of foods that sustain the growth of intestinal microorganisms might prevent the process by which translocation of potentially pathogen bacteria occurs [48]. In 1995, Gibson and Roberfroid [49, 50] introduced the concept of prebiotic as a nondigestible food ingredient that beneficially affects the host by selectively stimulating the growth and/or activity of one or a limited number of bacteria in the colon, and thus improves host health. Prebiotics, which are not digested in the small intestine, enter the colon as intact large carbohydrates that are then fermented by the resident bacteria to produce short-chain fatty acids. The nature of this fermentation and the resulting pH of the intestinal contents dictate proliferation of specific resident bacteria. For example, infants fed-breast milk containing prebiotics support increased proliferation of bifidobacteria and lactobacilli (probiotic), whereas formula-fed infants produce more enterococci and enterobacteria. Clear criteria have been established for classifying a food ingredient as a prebiotic. These criteria are (1) resistance to gastric acidity, to hydrolysis by mammalian enzymes, and to gastrointestinal absorption; (2) fermentation by intestinal microflora; (3) selective stimulation of the growth and/or activity of those intestinal bacteria that contribute to health and well-being. Presently there are only 2 food ingredients that fulfill these criteria, that is, inulin and transgalactooligosaccharides (TOS) [51]. The current most popular targets for prebiotic use are lactobacilli and bifidobacteria. Bifidobacteria are able to break down and utilize inulin-type fructans by the action of the b-fructofuranosidase enzyme. The mechanisms of action of prebiotics are complex and so far not yet well known or understood. These mechanisms are summarized below [52–60].

(1) Sources of carbon and energy for bacteria growing in the large bowel [precursors of short-chain fatty acids (SCFA): acetate, propionate, and butyrate] by saccharolytic fermentation [53, 55, 61]. (2) Immunological effects: (a) activate leukocytes in the gut-associated lymphoid tissue (GALT) system [56–58]; (b) increase cell numbers in Peyer's patches [57]; (c) enhance production of bacteriocins [59]; (d) enhance IgA levels in the small intestine and caecum [60]. (3) Improve gut-barrier function [57, 58]. (4) Acidify intestinal contents. (5) Improve bioavailability of calcium and magnesium [61, 62]. (6) Foster absorption of water and sodium [54]. (7) Promote gut transit.

## 6. Probiotics

Probiotics are commercially available microorganisms which, when ingested as individual strains or in combinations, offer potential health benefits to the host. These agents are often concurrently administered with substances that promote bacterial colonization and growth (prebiotics): in this instance, they are referred to as synbiotics [63]. The beneficial effects of probiotics in critically ill patients can be summarized as follows: (1) immunological effects, such as activation of leukocytes in the gut-associated lymphoid tissue system, increased cell number in Peyer's patches, enhanced immunoglobulin A levels [64], production of bacteriocins, inhibitory effect on proinflammatory cytokines tumor necrosis factor-alpha, interleukins IL-1 and IL-6, stimulation of anti-inflammatory cytokines (IL-10); (2) improved gut barrier function; (3) acidifying intestinal contents inhibition of pathogenic bacteria; (4) improved bioavailability of calcium and magnesium; (5) facilitated absorption of water and sodium; (6) increased intestinal motility. The effectiveness of probiotics is related to their ability to survive in the acidic and alkaline environment of stomach and duodenum, respectively, as well as their ability to adhere to the colonic mucosa and to colonize the colon [11]. Some probiotics, for example, *Lactobacillus *GG and *L. plantarum* 299v are better able to colonize the colon than others [11]. *Saccharomyces boulardii *are non-LA yeast and secret a protease causing proteolysis of Toxin A and Toxin B of *C. difficile* responsible for antibiotic-associated diarrhea (AAD) and, therefore, should be avoided [11]. Bifidobacteria are gram-positive anaerobic lactic acid bacilli (LAB), colonize the colon within days of birth and its population remains stable until advanced age. Lactobacilli are gram-positive, facultative anaerobic LAB, and are normal inhabitant of human gut. *L. plantarum* 299v adheres to the intestinal mucosa to reinforce its barrier function, thus prevents attachment of pathogens to the intestinal wall [65]. *Lactobacilllus *GG was found to eradicate *C. difficile* in patients with relapsing colitis. *L. plantarum* ST31 produces bacteriocins to limit the growth of potential pathogens. *L. casei* increases the level of circulating IgA. *L. acidophilus* and *B. bifidum* appear to enhance the phagocyte activity of circulating granulocytes. *Bacillus clausii* are gram-positive spore-forming strictly aerobic non-LAB and constitute less than 1% of gut microflora. *B. clausii* stimulates CD4 proliferation and lymphocytic activity in Peyer's patches. It also leads to increase in IgA-positive lymphocytes and HLA-DR positive T lymphocytes [66]. Multistrain probiotics seem to be better than single-strain ones, as individual probiotics have different functions and show synergistic effects when administered together [67]. Various single-strain and multistrain probiotics are commercially available for clinical use, majority being LABs.

## 7. Pre- and Pro Biotics in Critically Ill Children: Available Evidence

Circumstantial evidence suggests that probiotics alone or in combination with prebiotics are effective in preventing necrotizing enterocolitis (NEC), fungal colonization, and in improving feeding tolerance. They have also proved to be microbiologically safe and clinically well tolerated. To this regard, Manzoni reported a 6-year, two-center experience of routinary *Lactobacillus rhamnosus* GG (LGG) use in very low-birth-weight infants (3 × 10^9^ CFU/day, in single oral dose, since 4th day of life, for 4-to 6-week courses) [68]. No adverse effects or intolerances nor clinical sepsis attributable to LGG occurred. In a randomized controlled trial on 231 infants weighing 750–1499 g at birth, Braga et al. found that oral supplementation of human milk with *Bacillus breve* and *Lactobacillus casei* reduced the occurrence of NEC [69]. It was considered that an improvement in intestinal motility might have contributed to this result. Similarly, Bin-Nun showed in a randomized controlled trial that a probiotic mixture of *Bifidobacteria infantis*, *Streptococcus thermophilus*, and *Bifidobacteria bifidus* reduced the incidence and severity of NEC in very low-birth-weight neonates [70]. In a larger randomized, multicenter controlled trial, Lin et al. showed that *Bifidobacterium bifidum* and *Lactobacillus acidophilus*, added to breast milk or mixed feeding, twice daily for 6 weeks, reduced the incidence of death or NEC [71]. Taken together, these data favor the use of oral probiotics for the prevention of NEC in preterm infants. An improved outcome after treatment with *Lactobacillus reuteri* or *L. rhamnosus* was also observed in a group of 249 preterm infants by Romeo et al. who showed a reduced colonization by *Candida*, protection from late-onset sepsis and reduced abnormal neurological outcome [72]. In contrast with these positive results, Sari and Dani found no benefit in administering *Lactobacillus sporogenes* or LGG in reducing the incidence of NEC [73, 74]. Type of probiotics used, as well as the timing and dosage, may explain such differences. As compared to neonates, fewer studies have been published in older children admitted to intensive care, the majority of which focusing on safety and beneficial changes of intestinal flora. Simakachorn et al. randomized 94 patients between 1 and 3 years old who were requiring mechanical ventilation to receive either a test formula containing a synbiotic blend (composed of 2 probiotic strains (*Lactobacillus paracasei* NCC 2461 and *Bifidobacterium longum* NCC 3001), fructooligosaccharides, inulin, and Acacia gum, or a control formula. Infants in the test group tolerated well pre- and pro biotics [75]. Faecal bifidobacteria and total lactobacilli were higher in the test group, whereas enterobacteria levels diminished. The authors concluded that the enteral formula supplemented with synbiotics was well tolerated by children in intensive care units; it was safe and produced an increase in faecal bacterial groups. Honeycutt et al. evaluated the efficacy of probiotics in reducing the rates of nosocomial infection in 61 patients admitted to a pediatric intensive care unit [76]. Children were randomized to receive either one capsule of LGG or placebo once a day until discharge from the hospital. In this study, LGG was not shown to be effective in reducing the incidence of nosocomial infections. Similarly, a systematic review on 999 critically ill adults revealed no beneficial effect of probiotics/synbiotics in term of clinical outcomes, length of intensive care unit stay, incidence of nosocomial infection, pneumonia and hospital mortality [77]. In conclusion, it appears that beyond the neonatal age no clear evidence supports the use of probiotics in critically ill patients. Well-designed, large-scale, clinical trials are therefore needed to define optimal probiotic species, doses, and whether combination therapy is superior to single-agent therapy.

## 8. Conclusions

Bacterial translocation as a direct cause of sepsis is an attractive hypothesis that presupposes that in specific situations bacteria cross the intestinal barrier, enter the systemic circulation, and cause SIRS. Critically ill children are at increased risk for bacterial translocation, particularly in the early postnatal age. Predisposing factors include intestinal obstruction, obstructive jaundice, intra-abdominal hypertension, intestinal ischemia/reperfusion injury and secondary ileus, and immaturity of the intestinal barrier per se. Despite good evidence from experimental studies to support the theory of bacterial translocation as a cause of sepsis, there is no definite evidence in human studies to confirm that translocation is directly correlated to bloodstream infections in critically ill children. Besides, attempts at the use of pre- or pro-biotics have not always translated into clinical benefits to patient care, except for prevention of NEC in the neonatal population. Therefore, further research in this field is needed to help clinicians to make correct decisions concerning protection of the gut in the intensive care unit and to decide for possible therapeutic use of pre- and pro-biotics.
